# Chimeric Antibody Engineering Against *Bacillus anthracis* Lethal Toxin: Neutralization Efficacy and Mechanism of Action

**DOI:** 10.3390/toxins18010031

**Published:** 2026-01-09

**Authors:** Olga V. Kalmantaeva, Maksim A. Marin, Anastasia A. Ershova, Alena K. Ryabko, Yana O. Romanenko, Tatyana I. Kombarova, Ivan A. Dyatlov, Victoria V. Firstova

**Affiliations:** State Research Center for Applied Microbiology and Biotechnology, 142279 Obolensk, Russiaanastasiia.yershova@mail.ru (A.A.E.); ryabko_alena@mail.ru (A.K.R.); firstova@obolensk.org (V.V.F.)

**Keywords:** *Bacillus anthracis*, anthrax lethal toxin, protective antigen, monoclonal antibodies (mAbs), confocal laser scanning microscopy

## Abstract

*Bacillus anthracis* has three main virulence factors: an extracellular capsule and two binary toxins (lethal toxin—consists of a lethal factor and a protective antigen, and edema toxin—consists of an edema factor and a protective antigen). In the Russian Federation, the epidemiological situation regarding anthrax infection remains unfavorable. In the late stages of an anthrax infection, antibiotic therapy becomes ineffective and the patient dies within 24 h as a large amount of lethal toxin accumulates in the patient’s blood. Antibodies capable of neutralising lethal toxin (LT) can be an effective treatment for these patients. The objective of the study was to construct a chimeric monoclonal antibody targeting the protective antigen of the LT and to elucidate its mechanism of toxin neutralization. In this work, a chimeric monoclonal antibody (xi1E10) directed against the protective antigen was successfully produced. Both in vitro and in vivo experiments demonstrated the capacity of xi1E10 to neutralize lethal toxin. Confocal microscopy revealed that xi1E10 effectively suppresses the formation of a functional pore, thereby blocking the translocation of the lethal factor into the cytosol. These findings indicate that the monoclonal antibody xi1E10 represents a promising candidate for the development of a therapeutic drug.

## 1. Introduction

Anthrax is an anthropozoonotic infection caused by the Gram-positive, aerobic, spore-forming, rod-shaped bacterium *Bacillus anthracis*.

In 2024, cases of anthrax in humans have been identified in a number of countries in Africa, Asia, North and South America and Europe. A large number of cases of anthrax infection in humans are being recorded in Africa. In 2024, 487 cases of anthrax infection were recorded in Zimbabwe, 300 in Kenya, 211 in Uganda with 8 fatalities, and 163 in South Sudan. In 2024, in the West Department of Haiti (North America) 12 people fell ill with anthrax, two of whom were fatal. In the United States (Texas), one person was diagnosed with anthrax in 2024. In Colombia (South America), two people were diagnosed with anthrax. In August 2024, three people fell ill with anthrax in Albania (Europe) [1]. In the Russian Federation, the epidemiological situation of anthrax infection continues to be unfavorable. Sporadic cases of anthrax are reported; and there are more than 35,000 stationary anthrax-affected sites with risk factors, including approximately 8000 anthrax cattle burial sites. The highest concentration of these burial sites is found in the Volga, Central and Southern Federal Districts of the Russian Federation. In 2024, one anthrax outbreak was detected in Russia, with three human cases. However, the largest recent anthrax outbreak occurred in July-August 2016 in the Yamal region of the Yamalo-Nenets Autonomous district, which resulted in 36 people falling ill, including 18 children, and one fatal outcome [2].

*Bacillus anthracis* poses a bioterrorism threat, as evidenced by the 2001 U.S. attacks (16 cases, 5 fatalities from inhalational/cutaneous anthrax) and European heroin-related outbreaks (2009–2011; >50 cases with toxic shock) [3]. The latter predominantly resulted in fatalities due to toxic shock, necessitating combination therapy with antibiotics and monoclonal antibodies targeting PA and LF domains [4,5,6].

The genome of *Bacillus anthracis* comprises a single covalently closed chromosome and two virulence plasmids, pXO1 and pXO2, encoding the immunologically inert capsule and anthrax toxin, respectively. Its primary virulence factors include an extracellular capsule and two binary toxins: lethal toxin (lethal factor + protective antigen) and edema toxin (edema factor + protective antigen). Anthrax toxin, a tripartite A-B structure, features enzymatic A subunits—edema factor (EF; 89 kDa calmodulin-dependent adenylate cyclase) and lethal factor (LF; 90 kDa zinc metalloprotease)—and the receptor-binding/pore-forming B component, protective antigen (PA; 83 kDa precursor). PA engages two principal cell-surface receptors: TEM8 (ANTXR1) and CMG2 (ANTXR2).

**Figure 1:**
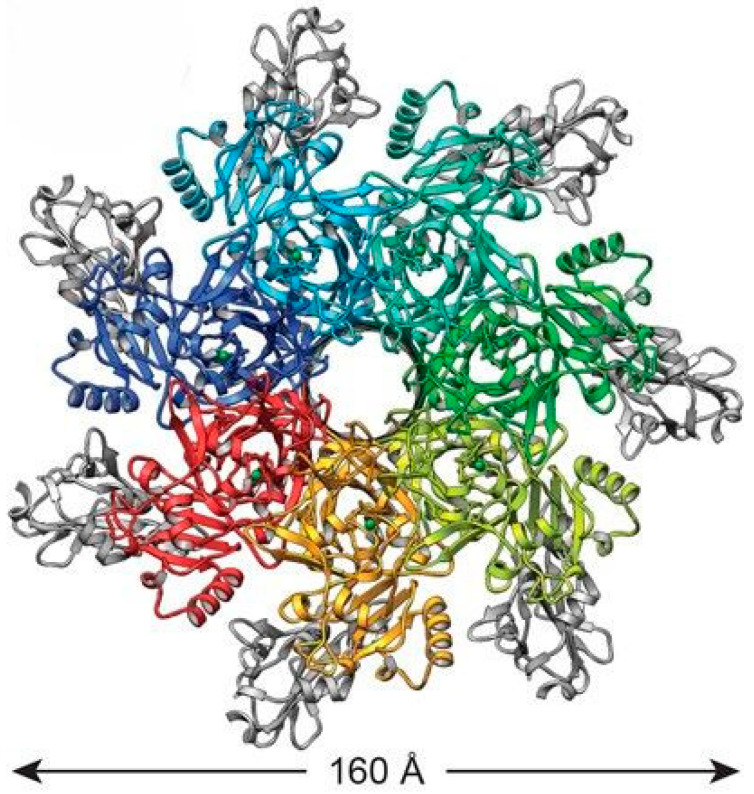
Atomic model of PA pores. The top view of the PA atomic model is depicted as ribbons. Domain IV is colored gray [8].

The 735-amino-acid protective antigen (PA) of *Bacillus anthracis* comprises four domains: domain 1 (residues 1–258) binds LF/EF; domain 2 (259–487) facilitates PA63 oligomerization and membrane insertion via a flexible loop forming a cation-selective channel [7]; domain 3 (488–595) aids oligomerization/LF-EF binding; and domain 4 (596–735) mediates receptor engagement with TEM8 (ANTXR1), CMG2 (ANTXR2), and β1-integrin. These von Willebrand factor type A-containing transmembrane receptors, differing primarily in cytoplasmic tails, undergo furin cleavage of PA83 to PA63 (removing 20 kDa N-terminal fragment), enabling PA63 prepore assembly (heptamer/octamer) independent of PA20. Analysis of the crystallographic structure of the heptameric prepore showed that it is built on the basis of a monomer-monomer contact between the surfaces of domains 1′ and 4 of PA63. Acidification triggers domain 2 reconfiguration—cup (2c) and stalk (2s)—forming a ~2-nm pore for unfolded LF/EF translocation (stoichiometries PA7LF3/PA8LF4), with LF/EF accelerating oligomerization (Figure 1).

PA–LF/EF complexes undergo clathrin-dependent endocytosis, requiring LRP6 co-engagement with CMG2/TEM8/β1-integrin; calpain-mediated talin degradation facilitates uptake. Endosomal acidification induces PA domain 2 insertion, forming a true pore for unfolded LF/EF translocation into cytosol. LF (94 kDa Zn^2+^-metalloprotease; 4 domains) cleaves N-terminal MAPKK sequences, disrupting immune signaling and macrophage viability via MKK-dependent/independent cytotoxicity.

PA_83_ binds to the cell surface receptor and is cleaved by cellular furin or a furin-like protease to generate an active, 63-kDa form (PA_63_). PA_63_ is self-assembled into a heptameric or octameric receptor-bound prepore, which contains high-affinity binding sites for EF and LF. The toxin-receptor complexes are then engulfed into the cell through a receptor-mediated, clathrin-dependent endocytosis, which requires post-translational modifications (e.g., ubiquitination and phosphorylation) on the receptor cytosolic domain. After uptake from the cell surface, PA undergoes conformational changes induced by the low pH in endosomes and forms pores in the endosomal membranes that act as a channel for the movement of enzyme subunits [9]. EF and LF enter the cytosol through a pore in the endosomal membrane. In the cytosol, EF increases cAMP levels and causes water retention in the cell, leading to edema, and LF cleaves mitogen-activated protein kinases (MAPKKs), leading to apoptosis, necrosis, cell hypoxia and ultimately death [9,10].

PA is the central component of the lethal toxin and consists of 4 structurally independent domains. The main function of domain I is to bind PA to LF. Domain II is involved in the formation of a loop that is subsequently embedded in the membrane and forms a cation-selective channel. Domain III is involved in the oligomerization of PA and the binding of the oligomeric structure to LF and/or EF. The function of domain IV is to bind PA to receptors on the surface of eukaryotic cells [11]. The protective antigen (PA) elicits an immune response, and all contemporary acellular or attenuated live anthrax vaccines either contain or express PA [12].

In the late stages of an anthrax infection, antibiotic therapy becomes ineffective and the patient dies within 24 h as a large amount of lethal toxin accumulates in the patient’s blood, leading to the development of shock and death. Antibodies capable of neutralising LT can be an effective treatment for these patients.

Active development of toxin-neutralizing monoclonal antibodies (mAbs) yielded raxibacumab (FDA-approved 2012), a fully human IgG1λ mAb targeting PA to preclude receptor binding, effective solely pre-oligomerization [13]. In cynomolgus monkeys, single-dose raxibacumab boosted aerosol anthrax survival to 64% [14]. Safety was confirmed in 326 healthy volunteers, but clinical trials of therapeutic efficacy in human have not been conducted. Obiltoxaximab (Anthim^®^, ETI-204; FDA 2016), a chimeric IgG1κ mAb (148 kDa), similarly neutralizes PA for inhalational anthrax prophylaxis/treatment [15]. Anthrasil, plasma-derived human IgG from BioThrax-immunized donors, complements antibiotics by engaging diverse PA epitopes post-receptor binding [16,17].

Despite these advances, limitations persist: neither penetrates the blood-brain barrier for meningeal anthrax, Anthim risks hypersensitivity/anaphylaxis (premedication advised), and both require refrigeration (2–8 °C), IV administration (IM preferred in mass-casualty scenarios), with rare allergic reactions. Treatment with antibiotics alone is effective for four days after aerogenic inoculation with *B. anthracis* spores. The use of antitoxin in combination with antimicrobials extends the window of effective treatment to one week after *B. anthracis* infection [18].

Previously, our team generated mouse monoclonal antibodies (mAbs) that effectively neutralize LT [19]. Among the hybridoma clones generated, clone 1E10 demonstrated the greatest potential, producing antibodies specific to domain IV of the *Bacillus anthracis* protective antigen (PA). Murine monoclonal antibodies (mAbs) are unsuitable for human therapy due to their reactogenicity and immunogenicity; however, they can serve as a basis for chimeric mAbs, which incorporate only 20–25% murine protein sequences.

The objective of this study was to develop a chimeric monoclonal antibody targeting the anthrax protective antigen and to elucidate its mechanism of lethal toxin neutralization.

## 2. Results

### 2.1. Production and Characterization of Chimeric Monoclonal Antibodies to the Protective Antigen

The murine hybridoma 1E10 served as the source for the development of the chimeric monoclonal antibody. Earlier studies characterized mAb 1E10, demonstrating its strong neutralizing efficacy against anthrax lethal toxin in both in vitro assays and in vivo mouse models [9].

**Figure 2:**
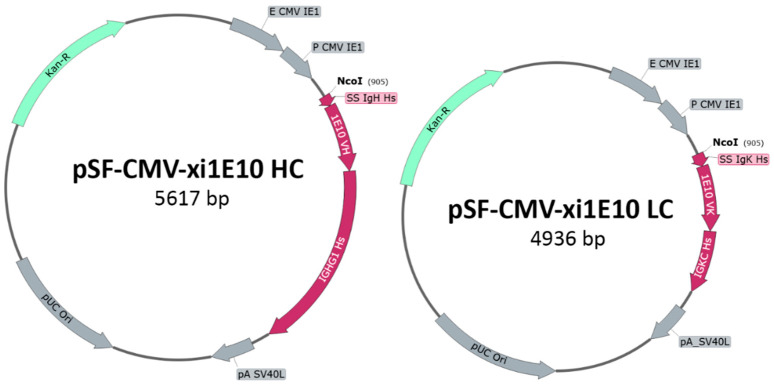
Plasmid maps of pSF-CMV-xi1E10_LC and pSF-CMV-xi1E10_HC for xi1E10 mAb expression.

**Figure 3:**
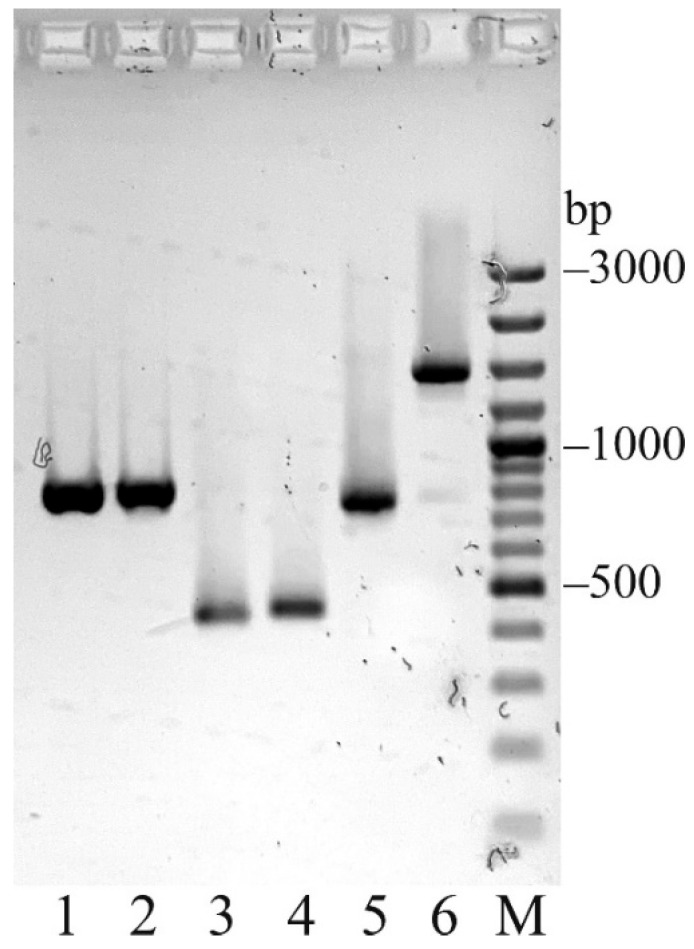
PCR amplification of 1E10 cDNA fragments and open reading frames (ORFs) of xi1E10 light (LC) and heavy chains (HC). Lanes: 1—1E10 LC; 2—1E10 HC (Fd region); 3—1E10 VK with signal peptide for molecular cloning; 4—1E10 VH with signal peptide for molecular cloning; 5—xi1E10 LC ORF from pSF-CMV-xi1E10 HC; 6—xi1E10 HC ORF from pSF-CMV-xi1E10 HC; M—GeneRuler 100 bp Plus DNA Ladder (Thermo Fisher, Vilnius, Lithuania).

The DNA constructs expressing the light and heavy chains of xi1E10 were obtained by generating plasmid vectors pSF-CMV-xi1E10_LC and pSF-CMV-xi1E10_HC (Figure 2), respectively. The cDNA fragments of the murine mAb (lanes 1–4) and the resulting recombinant DNA chains of the chimeric mAb (lanes 5, 6) after PCR amplification are shown in Figure 3.

**Figure 4:**
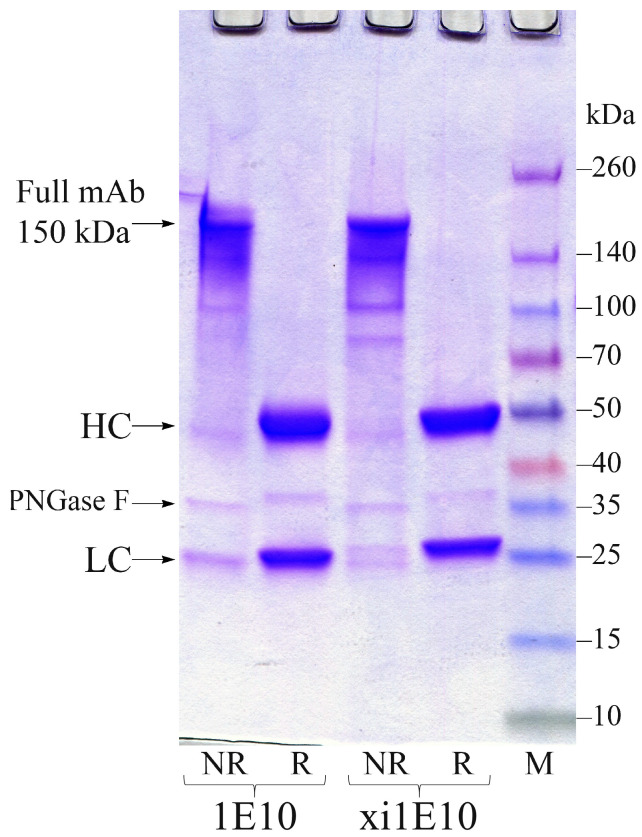
SDS-PAGE analysis of purified mAbs under reducing (R) and non-reducing (NR) conditions. Lanes were loaded with 8 μg of protein. NR = Non-Reduced; R = Reduced; M—Spectra Multicolor Broad Range Protein Ladder (Thermo Fisher, Vilnius, Lithuania).

For the expression of mAb xi1E10, plasmids pSF-CMV-xi1E10_LC and pSF-CMV-xi1E10_HC were co-transfected into ExpiCHO-S cell culture. The mAb xi1E10 was purified from culture supernatant, while mAb 1E10 was purified from ascites fluid, both by affinity chromatography followed by gel filtration. The yield of purified 1E10 antibody was ~5 mg per ascites fluid preparation. Approximately 17 mg of xi1E10 was obtained from 1 L of culture after purification. The purity of protein products pre-treated with PNGase F (NEB, Ipswich, MA, USA), was assessed by SDS-PAGE under reducing and non-reducing conditions in 4–20% Mini-PROTEAN TGX Gel (Bio-Rad, Hercules, CA, USA) (Figure 4). Analysis of the full-length mAbs by SDS-PAGE under non-reducing conditions revealed no dimeric or aggregated species. However, several minor bands were detected, most likely corresponding to antibody fragments with partially reduced interchain disulfide bonds: 2 HC + 1 LC (125 kDa), 2 HC (100 kDa), 1 HC + 1 LC (75 kDa) and free HC/LC (50/25 kDa). The presence of these forms is not indicative of protein degradation in the sample. Their formation is most likely an artifact caused by either the lateral diffusion of β-mercaptoethanol from adjacent wells containing reduced samples during the gel loading step, or trace amounts of reducing agent present in the PNGase F enzyme used for prior deglycosylation. Both reduced samples of 1E10 and xi1E10 were pure.

**Figure 5:**
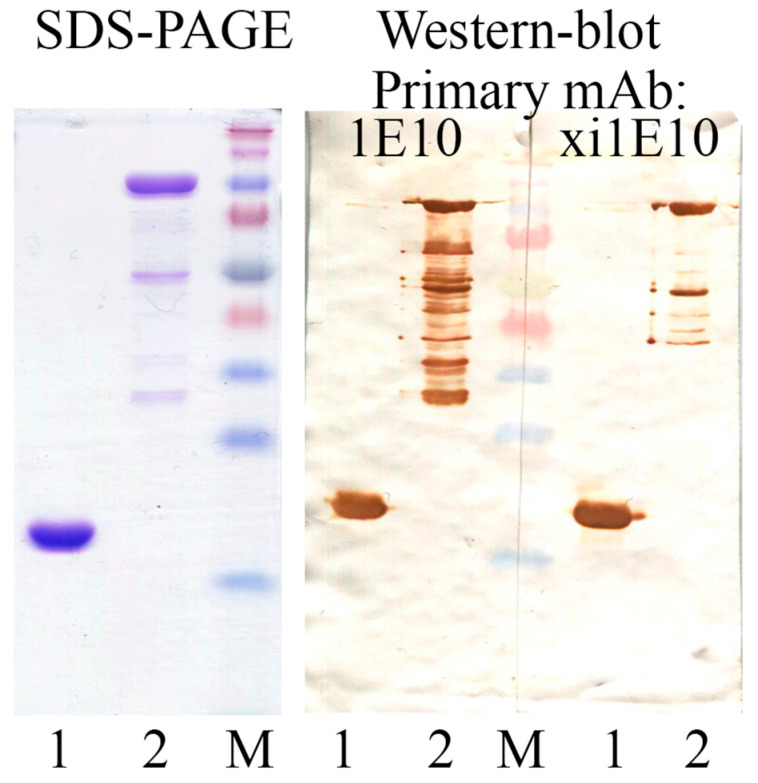
SDS-PAGE of target antigens (**left**) and Western blot (**right**) using studied mAbs as primary antibodies. Lanes were loaded with 8 μg of protein for staining and 0.2 μg for blotting. 1—rPA IV; 2—rPA; M—Spectra Multicolor Broad Range Protein Ladder.

The activity of the purified mAbs was qualitatively evaluated by Western blot with the target antigens rPA and rPA IV (Figure 5).

**Figure 6:**
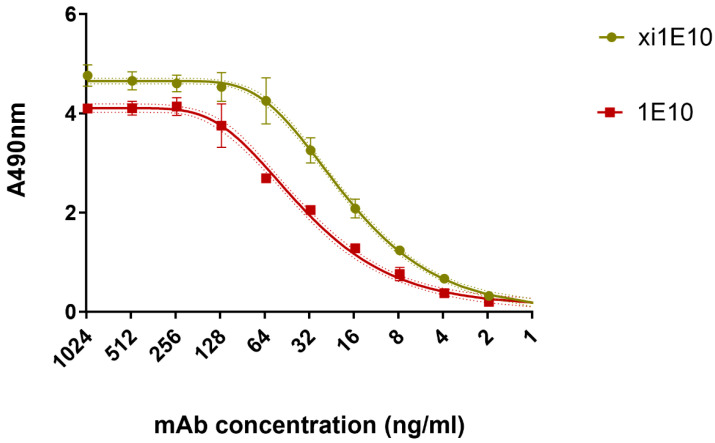
ELISA binding curves of mAbs at serial dilutions. Error bars represent 95% confidence intervals.

A semi-quantitative assessment of antibody binding in serial dilutions to rPA IV antigen was performed by ELISA (Figure 6). Some differences in signal intensity were attributed to the use of different HRP-conjugated secondary antibodies, but it was evident that both antibodies bound the antigen with high affinity.

**Figure 7:**
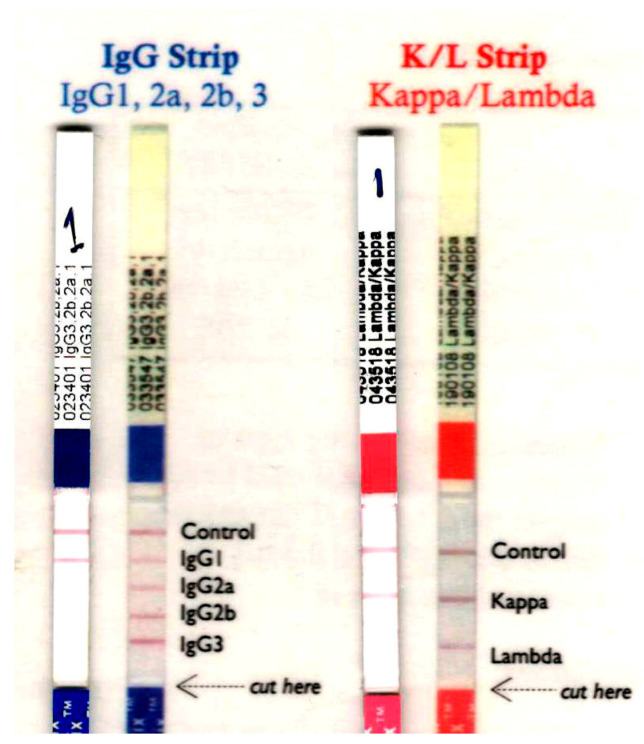
Class specificity of the mouse mAb 1E10.

Immunochromatographic analysis identified 1E10 as an IgG1 isotype with a κ light chain (Figure 7).

Affinity parameters of mAb 1E10 and xi1E10 interactions with rPA IV were quantified by surface plasmon resonance, yielding equilibrium dissociation constants. The equilibrium dissociation constants K_d_ for the mAbs were determined as follows: K_d_1E10 = 4.3 × 10^−8^ M, Kdxi1E10 = 1.1 × 10^−7^ M.

**Figure 8:**
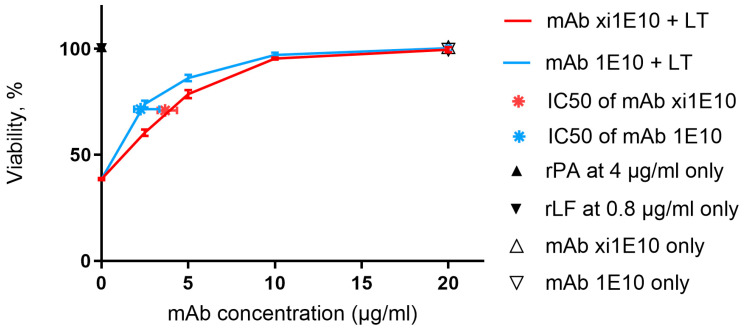
In vitro toxin-neutralizing activity assessed by MTT assay on J774A.1 cells. Error bars indicate standard deviation. Vertical error bars represent SEM. Horizontal error bars for IC50 values represent the 95% confidence interval.

The in vitro toxin-neutralizing activity was analyzed using the J774A.1 cell line in an MTT assay. Lethal toxin neutralization was determined by measuring the increase in cell monolayer viability with increasing antibody concentrations in the presence of a fixed toxin dose. Analysis of dose-dependent neutralization demonstrated that mAb 1E10 exhibited higher neutralizing activity in vitro: its IC50 was 2.26 µg/mL, whereas the IC50 of mAb xi1E10 was 3.67 µg/mL. The comparison of fits was statistically significant (*p* < 0.001), corresponding to approximately a 1.6-fold potency advantage of mAb 1E10 (Figure 8).

**Figure 9:**
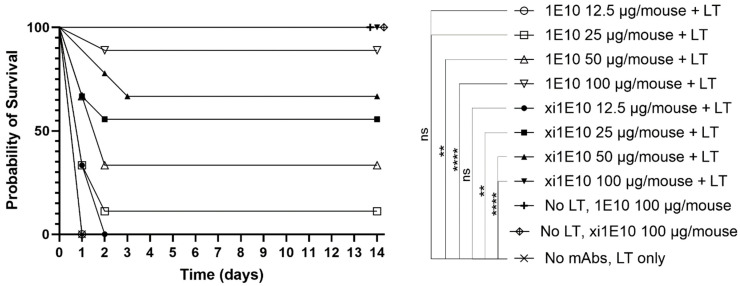
In vivo toxin-neutralizing activity in a mouse model. Statistically significant differences in mouse survival between experimental groups were assessed using the Mantel-Cox log-rank test. ** *p* < 0.01, **** *p* < 0.0001, ns (not significant) *p* > 0.05.

Subsequently, the in vivo toxin-neutralizing activity of the mAbs was assessed in a murine model. Comparing the survival curves of mice in the experimental groups (Figure 9), it was evident that both mAbs exhibited neutralizing activity in vivo, and significantly protected mice from LT-induced death. However, no statistically significant difference in survival was observed between mice treated with the analyzed doses of xi1E10 and 1E10.

### 2.2. Study of the Mechanism of Neutralization of mAb xi1E10

In this study, we investigated the underlying mechanisms by which xi1E10 neutralizes lethal toxin. Since this mAb was obtained to the IV domain of the lethal toxin PA, in the first stage of the study we assessed its effect on LT internalization in J774A.1 murine macrophage cells using confocal laser scanning microscopy.

**Figure 10:**
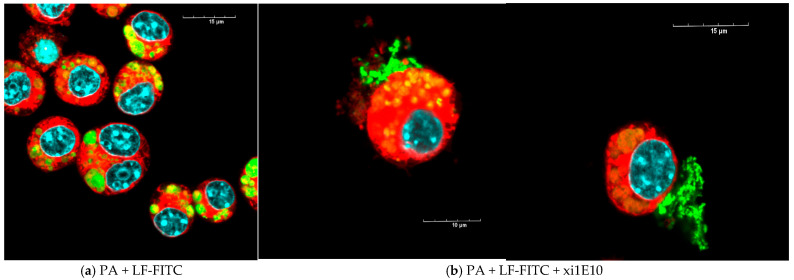
(**a**) J774A.1 cells after 1.5 h of incubation with FITC-labeled lethal anthrax toxin (PA + LF-FITC—green). Cytoplasm is stained with CellTracker (red); nuclei—Hoechst 33258 (blue). Scalebar is 15 μm. (**b**) J774A.1 cells after 1.5 h of incubation with a complex of FITC-labeled lethal anthrax toxin (PA + LF-FITC—green) and monoclonal antibody to protective antigen 1E10. Cytoplasm is stained with CellTracker (red); nuclei—Hoechst 33258 (blue). Scalebar is 10 μm on the left image, 15 μm on the right image.

**Figure 11:**
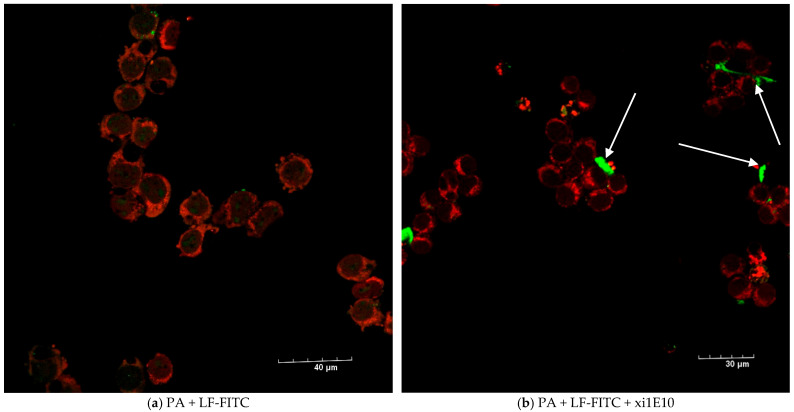
(**a**) J774A.1 cells after 1.5 h of incubation with FITC-labeled lethal anthrax toxin (PA + LF-FITC—green). Late endosomes are labeled with polyclonal antibodies to Rab7 (red) Scalebar is 40 µm. (**b**) J774 cells after 1.5 h of incubation with a complex of FITC-labeled lethal anthrax toxin (PA + LF-FITC—green) and monoclonal antibody to protective antigen xi1E10. Late endosomes are labeled with polyclonal antibodies to Rab7 (red). White arrows point to lethal toxin on the cell surface. Scalebar is 30 µm.

The lethal toxin (without of mAb xi1E10) was completely internalized into the cells after 1.5 h and localized in rounded compartments (Figure 10a); we did not detect any toxin on the cell surface (Figure 11a). After preliminary incubation of anthrax LT with mAb xi1E10, most of the toxin was on the cell surface and could not be internalized into the cells (Figure 10b and Figure 11b, white arrows).

**Figure 12:**
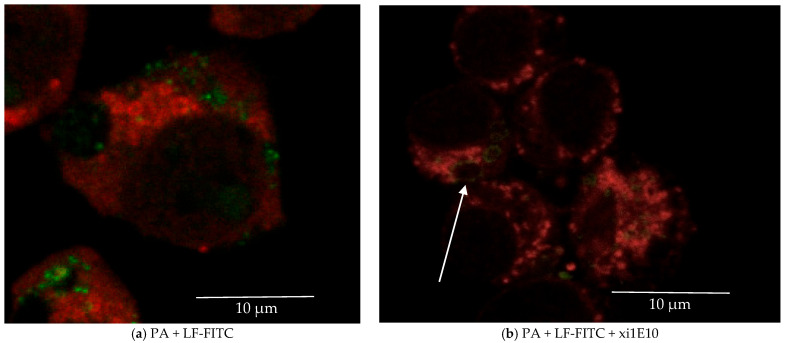
(**a**) J774A.1 cells after 1.5 h of incubation with FITC-labeled lethal anthrax toxin (PA + LF-FITC—green). Late endosomes are stained with polyclonal antibodies to Rab-7 (red). (**b**) B. J774 cells after 1.5 h of incubation with a complex of FITC-labeled lethal anthrax toxin (PA + LF-FITC—green) and monoclonal antibody to the protective antigen xi1E10. Late endosomes are stained with polyclonal antibodies to Rab-7 (red). The white arrow points to lethal toxin localized in round hollow structures. Scalebar is 10 µm.

Nevertheless, some LT was internalized into the cell. Therefore, we investigated the subsequent intracellular trafficking of LT. Given that the PA forms a pore in the membrane of late endosomes, we determined whether LT is localized in late endosomes. Late endosomes were visualized using antibodies against the late endosome receptor Rab-7 conjugated to Alexa Fluor 647. Our observations indicated that following pre-incubation with mAb xi1E10, LT was absent from late endosomes; instead, the toxin localized to distinct ring-shaped hollow vesicles lacking Rab-7 expression (Figure 12b, white arrow). In contrast, LT without antibody treatment accumulated in the cytosol, and its localization was not detected in late endosomes.

**Figure 13:**
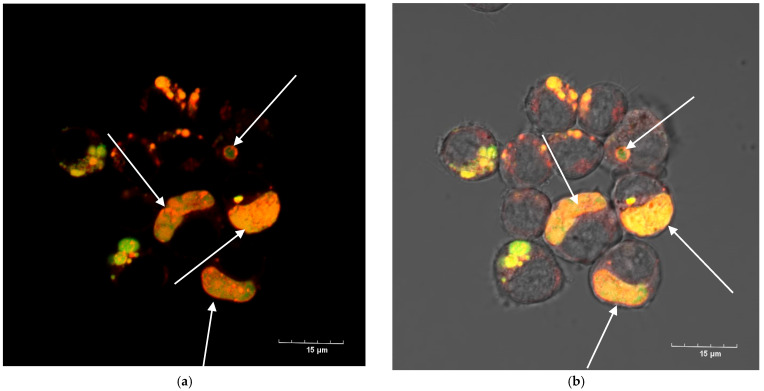
J774A.1 cells after 1.5 h of incubation with a complex of FITC-labeled lethal anthrax toxin (PA + LF-FITC—green) and a monoclonal antibody to the protective antigen xi1E10. Lysosomes are stained with Lysotracker Red DND 99 (red). (**a**) Confocal image is shown in the green and red channels. (**b**) Confocal image is shown in the green, red and transmitted light channels. The white arrows points to lethal toxin localized in lysosomes. Scalebar is 15 µm.

Based on these observations, we hypothesized that xi1E10 facilitates the translocation of lethal toxin to lysosomes for degradation. To investigate this, lysosomes were stained with the fluorescent dye Lysotracker Red DND 99. Colocalization of FITC and Lysotracker Red DND 99 fluorochromes showed that toxin-xi1E10 complexes were localized in lysosomes. The Pearson correlation coefficient was 0.779, indicating a true colocalization. These data confirm that LT after pre-incubation with mAb xi1E10 is localized in lysosomes (Figure 13, white arrows). Toxin-xi1E10 complexes accumulate in lysosomes, where they are likely subjected to proteolytic degradation, thereby providing the neutralizing effect of mAb xi1E10.

## 3. Discussion

The efficacy of monoclonal antibodies to LT in the treatment of anthrax infection has been demonstrated at various stages of the disease. The majority of studies have focused on the acquisition of antibodies against PA B. anthracis, given the key role of PA in the pathogenesis of anthrax. PA ensures the penetration of LT and OT into the cell, so inhibition of PA provides protection of cells from the toxic effects of both toxins. In addition, it is the binding of PA to receptors on the surface of a eukaryotic cell (TEM8/ANTXR1, CMG2/ANTXR2) that is the first event in the multi-stage intracellular penetration of the *B. anthracis* toxin.

Currently, there are a number of mAbs to the receptor-binding domain IV of PA: Raxibacumab [13]; IQNPA [20]; Obiltoxaximab ETI-204 (Anthim^®^) [15]; W1 [21]. Of these, only Raxibacumab and Obiltoxaximab have passed all stages of clinical trials and are recommended by the FDA together with antibiotics for combination therapy of inhalation anthrax. The remaining antibodies have shown a good therapeutic effect in animals (Table 1) [21]. Nevertheless, the development and production of highly neutralizing and humanized mAbs to LT remain urgent tasks for researchers.

Monoclonal antibodies Raxibacumab, IQNPA, Obiltoxaximab (ETI-204, Anthim^®^), and W1 neutralize *Bacillus anthracis* lethal toxin primarily by targeting the protective antigen (PA), specifically its receptor-binding domain IV, thereby blocking toxin entry into host cells. Raxibacumab and Obiltoxaximab have well-established profiles with FDA approval, demonstrating in vitro and in vivo efficacy, including confirmed blockade of PA–CMG2 binding and toxin internalization. IQNPA is presumed to function similarly by targeting domain IV, although detailed mechanistic data are relatively sparse. W1 shares this common receptor-binding inhibition mechanism but differs in epitope recognition and affinity, with less extensive characterization available [21].

Previously, mAbs1E10, which specifically targets domain IV of the *Bacillus anthracis* protective antigen (PA), were developed in our laboratory. This antibody exhibited strong protective efficacy in a mice model [19]. Using genetic engineering techniques, the chimeric antibody xi1E10 was constructed by combining the murine variable regions of the 1E10 antibody with human constant regions. This design aimed to preserve the antigen specificity and neutralizing capacity of the original murine 1E10 while reducing immunogenicity in humans.

Immunochromatographic analysis confirmed that both the parental murine mAb 1E10 and the chimeric xi1E10 belong to the IgG1 isotype with a κ light chain. For monoclonal antibody-based therapeutics aimed at toxin neutralization, the IgG1 subclass is preferred due to its favorable pharmacokinetic profile and robust functional properties, as evidenced by approved antitoxins targeting botulinum and anthrax toxins. Notably, IgG1 exhibits high affinity for Fcγ receptors (FcγRI, FcγRIIa, FcγRIIIa), thereby mediating potent effector functions, including antibody-dependent cellular cytotoxicity (ADCC), antibody-dependent cellular phagocytosis (ADCP), and complement-dependent cytotoxicity (CDC) [22,23]. These effector mechanisms augment toxin neutralization by recruiting immune cells, a process essential for anthrax toxin, where antigen binding alone proves insufficient.

We evaluated the affinity of xi1E10, its toxin-neutralizing potency in vitro and in vivo, and the underlying neutralization mechanisms. Comparative analysis of the two mAbs revealed that the murine 1E10 exhibited superior affinity and greater in vitro neutralizing activity on a murine cell line. However, no statistically significant differences were observed in vivo toxin-neutralizing efficacy between the two mAbs in a mouse model. The superior affinity of murine mAb 1E10 for PA suggests enhanced in vivo neutralizing activity. However, the human IgG1 constant region in chimeric Mab exhibits markedly higher affinity for the murine neonatal Fc receptor (FcRn) compared to murine IgG1, thereby substantially prolonging the circulation time of the chimeric xi1E10 antibody relative to its parental counterpart and be the main reason for the equivalent in vivo protection of mAb 1E10 [24].

Ultimately, the therapeutic efficacy of toxin-neutralizing monoclonal antibodies is governed not only by the Fab region’s affinity for the toxin but also by the Fc region’s affinity for FcRn, which regulates antibody half-life (T1/2) and systemic exposure, quantified as the area under the concentration-time curve (AUC) in plasma, reflecting total drug exposure [25]. The chimeric mAb xi1E10 may also stimulate host immune responses upon formation of antigen-antibody complexes. Although evaluation of its effector functions (ADCC, ADCP, CDC) is feasible in mice, such studies are constrained by interspecies discrepancies in Fc receptors and complement pathways. Nevertheless, toxin-antibody complexes are cleared more efficiently by the immune system through phagocytosis activation, complement-mediated opsonization, and Fc receptor-mediated uptake by the reticuloendothelial system [22].

To evaluate the toxin neutralization activity in vitro, we used the macrophage-like cell line J774A.1. The rationale for choosing the cell line is that macrophages play a key role in B. anthracis-mediated pathogenesis. After infection, *B. anthracis* spores are engulfed by macrophages and induce the production of TNF-α and IL-6; components of the vegetative form are recognized by macrophages and stimulate the innate immune response [26,27]. In addition, studies investigating the in vitro efficacy of FDA-approved mAbs to anthrax toxin, such as Obiltoxaximab, also used a mouse macrophage cell line [15].

Although a murine macrophage cell line was chosen, we posit that these findings will translate effectively to human cells. Dekkers G. et al. demonstrated that human IgG subclasses exhibit comparable relative affinities for murine Fcγ receptors as for their human orthologs. Notably, human IgG binds murine FcγRs with remarkably high affinity—similar to that of murine IgG—and largely analogous to human IgG binding to human FcγRs, with only marginal reductions in affinity. These observations indicate that murine models effectively recapitulate FcγR-mediated effector functions of human IgG1 [28].

Monoclonal antibodies xi1E10, similarly to the antibodies presented above (Table 1), recognize domain IV of the protective antigen (PA), leading to the hypothesis that these antibodies inhibit the internalization of lethal toxin (LT) into the cell. In a previous study [4], potential inhibition steps of LT cytotoxicity were analyzed. Unexpectedly, monoclonal antibody 1E10 did not inhibit the following processes: the interaction of PA with the membrane of the J774A.1 macrophage-like cell line; the formation of oligomeric PA structures and prepore formation; the interaction between LF and PA; nor LT endocytosis in the presence of 1E10 antibodies. However, 1E10 antibodies inhibited the enzymatic activity of LF towards MEK1 and MEK2. Based on these findings, it was hypothesized that the mechanism of LT inhibition by monoclonal antibodies 1E10 is associated with disruption of functional pore formation and the inability of LF to translocate into the cytosol.

Confocal microscopy is preferred for elucidating the mechanisms of monoclonal antibody neutralization of toxins due to its superior spatial resolution and ability to generate high-contrast, three-dimensional images at the subcellular level. This technique allows precise visualization of toxin localization and trafficking within cells, enabling differentiation between various intracellular compartments such as early and late endosomes, lysosomes, and the cytosol.

In this study, we conducted more detailed investigations using confocal laser scanning microscopy and found that the monoclonal antibody xi1E10 inhibits the internalization of most part of LT into cells. A small fraction of LT bound to xi1E10 that did enter the cell was unable to translocate into the cytosol and accumulated in lysosomes, where it underwent proteolytic degradation. Collectively, these data indicate that xi1E10 neutralizes LT cytotoxicity by partially inhibiting LT entry into the cell and completely preventing true pore formation, thereby blocking the translocation of lethal factor (LF) into the cytosol and its subsequent enzymatic activity.

The mechanism of neutralization of anthrax toxin by Raxibacumab and Obiltoxaximab is similar [13,29]. The molecular dynamics of Raxibacumab’s interaction with the CMG2/TEM8 cellular receptors is based on steric hindrance: the Fab fragment of the antibody occupies a key PA epitope (the receptor-binding region), preventing the formation of the pre-pore oligomer PA63 and the subsequent translocation of lethal (LF) and edema (EF) factors into the cytosol. Raxibacumab binding does not affect PA83 → PA63 proteolysis, but it does block oligomerization and endosytosis by halting pH-induced conformational transitions (His121 protonation in endosomes) required for β-pore formation. The xi1E10 antibody stabilizes the inactive conformation of PA, inhibiting pH-dependent proteolytic maturation. Consequently, the β-loop of domain II fails to release and form the translocation channel. LF remains sequestered in the endosomal lumen, where it undergoes lysosomal degradation rather than cytosolic release, ultimately exhibiting no enzymatic activity due to N-terminal hydrolysis of its MAPKK substrate.

Currently available monoclonal antibodies targeting anthrax toxins exhibit several limitations, including suboptimal efficacy, specificity, or manufacturability. Consequently, the design and production of novel, highly effective mAbs against these toxins remain a pressing priority in biodefense and therapeutic research. Our synthesized mAb xi1E10 offers a valuable contribution to advancing studies in this critical field.

## 4. Conclusions

A chimeric monoclonal antibody xi1E10 was obtained. In vitro and in vivo experiments confirmed its ability to neutralize *B. anthracis* LT. This mAb is characterised by its high affinity, making it a promising candidate for the further development of a drug capable of neutralizing anthrax toxin. The neutralizing effect of the monoclonal antibody xi1E10 involves partially inhibiting the internalization of the lethal toxin into the cell and preventing the formation of a true pore in late endosomes. This promotes the complete degradation of the toxin in lysosomes and prevents its release into the cell’s cytosol.

## 5. Materials and Methods

### 5.1. Production and Purification of Recombinant Lethal Toxin Components

The recombinant proteins comprising *B. anthracis* lethal toxin—full-length protective antigen (rPA), lethal factor (rLF), and domain IV of PA (rPA IV), each containing an N-terminal Myc epitope and hexahistidine tag (6 × His)—were expressed in a prokaryotic system [16,17].

### 5.2. Generation and Purification of Monoclonal Antibodies Against Bacillus anthracis Protective Antigen

The hybridoma producing monoclonal antibody 1E10 was grown in vivo as ascites fluid in mice. The murine hybridoma, which is the producer of monoclonal antibody 1E10, was deposited at the State Collection of Pathogenic Microorganisms (Obolensk, Moscow region, Russia) on 2 September 2019, under accession number H-89. The murine antibody was isolated from ascites fluid by affinity chromatography using Protein G Sepharose (GE Healthcare, Little Chalfont, Buckinghamshire, UK), followed by additional purification on a Superdex 200 (GE Healthcare, UK) column.

Total RNA was extracted from the 1E10 hybridoma cells, followed by reverse transcription using the RevertAid RT Kit (Thermo Fisher, Vilnius, Lithuania). The sequences encoding the variable regions of mAb 1E10 were determined by the 5′-RACE method [30] with primer pairs MOCG12FOR/XSCTnTag and CKMOsp/XSCTnTag for amplification of Fd (VH + CH1) and LC (VK + CK) regions, respectively, using the first-strand cDNA template. The PCR products were cloned into the pUC19 plasmid. The resulting plasmids were sequenced to determine the VH and VK coding sequences. These regions were then amplified by PCR using primers that introduced NcoI restriction site and the H5/L1 signal peptide [31] DNA at the 5′ end, and BseRI site at the 3′ end for seamless fusion with the constant region DNA. The resulting products were cloned into the plasmid vectors pSF-CMV-HuIgG1_HC (OG527) and pSF-CMV-HuKappa_LC (OG528) from the Human IgG Vector Set (PP2409, OXGENE, Oxford, UK) to generate the heavy- and light-chain plasmids for xi1E10 expression: pSF-CMV-xi1E10_HC and pSF-CMV-xi1E10_LC. Recombinant xi1E10 was produced in ExpiCHO-S cells (Gibco, Jenks, OK, USA) following co-transfection with HC/LC plasmids at a 1:2 ratio. Pre-culture, transfection, and expression were performed according to the “Max Titer Protocol” from the user’s guide of ExpiCHO Expression System Kit (Thermo Fisher, Vilnius, Lithuania). The chimeric antibody was purified from the cell culture supernatant by affinity chromatography on Protein A Sepharose (GE Healthcare, UK), followed by size-exclusion chromatography with Superdex 200.

The immunological specificity of purified murine and chimeric mAbs toward rPA IV was assessed by ELISA. Antigen binding to both rPA and rPA IV was further verified by Western blot analysis. Secondary antibodies included: Anti-Mouse IgG (whole molecule) HRP (#A9044, RRID: AB_258431, Sigma-Aldrich, St. Louis, MO, USA) or Anti-Human IgG (γ-chain specific) HRP (#A6029; RRID: AB_258272, Sigma-Aldrich).

### 5.3. Determination of mAb Affinity Parameters

The equilibrium dissociation constant (K) for 1E10 and xi1E10 antibodies to the recombinant PA4d protein was determined by surface plasmon resonance (SPR) on a Biacore X100 instrument (Cytiva, Uppsala, Sweden). A HisTag-containing molecule capture chip (NTA-Au chip, iClueBio, Ansan-si, Gyeonggi-do, South Korea) was used. HBS-EP (10 mM Hepes, 150 mM NaCl, 3 mM EDTA, 0.005% Tween-20) pH 7.4 was used as the working buffer. The recombinant PA IVd protein was applied to the chip surface as a ligand at a concentration of 10 μg/mL at a flow rate of 10 μL/s for 60 s. The analyte solution (the studied antibodies 1E10 or xi1E10) was used at concentrations from 800 nM to 9.88 nM with a threefold titration step. The analyte was applied under the following conditions: the application rate was 30 μL/s, the application time was 120 s, and the dissociation time was 600 s. The chip surface was regenerated with a solution of 350 mM EDTA pH 8.0 for 30 s. The obtained sensorgrams were processed using Biacore X100 Evaluation Software v.2.0.2 (Cytiva, Uppsala, Sweden, Sweden). The sensorgrams were processed and calculated using Equilibrium Dissociation Constant.

### 5.4. Toxin-Neutralizing Activity of mAbs In Vitro

The toxin-neutralizing activity was assessed using the MTT test on the J774A.1 macrophage cell line, obtained from ATCC (TIB-67) Amoebae cultures Collection of Institute of Cytology RAS (St. Petersburg, Russia). A total of 2 × 10^4^ cells per well were seeded in 90 µL of growth medium into a 96-well plate and incubated overnight at 37 °C and 5% CO_2_.

The final concentrations of the lethal toxin (LT) components were: rPA—0.5 µg/mL, rLF—0.1 µg/mL. The antibody concentrations in the assay were 0, 2.5, 5, 10 and 20 µg/mL, corresponding to molar ratios of mAb:rPA of 0:1, 0.37:1, 0.73:1, 1.46:1, and 2.93:1. LT and mAb were pre-incubated for 1 h in PBS buffer., after which 10 µL of the mixture was added to the cells. For controls, 10 µL of PBS was added to wells intended to maintain 100% viability, while 10 µL of 0.25% thimerosal was added to wells designated for complete cell death. All samples were incubated with the cells for 4 h. After incubation, MTT solution was added to all wells, followed by 4 h incubation under the same conditions. The formazan crystals were then dissolved in DMSO, and absorbance was measured at 540 nm. The experiments were carried out in triplicate. Dose–response curves were fitted using a four-parameter logistic nonlinear regression model in GraphPad Prism 6.00. The mAb concentrations were plotted on a logarithmic scale. The response at zero antibody concentration (Bottom) was constrained to the value corresponding to cell viability in the presence of LT only. IC50 values were obtained from the fitted curves as the antilogarithm of the LogIC50 parameter.

### 5.5. Assessment of mAb Toxin-Neutralizing Activity in a Murine Model

Inbred BALB/c (H2^d^) mice (Stolbovaya Branch of the Federal State Budgetary Scientific Institution Scientific Center for Biomedical Technologies of the Federal Medical and Biological Agency of Russia) were used in the experiments. Mice weighed 18–20 g and were 6–8 weeks old, and were female. To determine the protective dose conferring lethal toxin (LT) neutralization, BALB/c mice (9 animals in each group) received mAbs intraperitoneally at doses of 12.5, 25, 50, or 100 μg per mouse. The control group received PBS. At 24 h post-immunization, animals were administered intravenously with 4LD50 of LT (50 μg rPA + 50 μg rLF). Mortality was recorded daily for 14 days. Statistically significant differences in mouse survival between experimental groups were assessed using the Mantel-Cox log-rank test.

The plan and procedure for conducting experiments using laboratory animals are presented in research protocol № VP-2025/3 “Evaluation of the ability of mouse monoclonal antibodies to neutralize the effect of the lethal toxin of *Bacillus anthtacis*”. All work with animals was carried out in accordance with the rules and regulations described in the sanitary and epidemiological rules and regulations 3.3686-21 [32], GOST (State Standard, Russian National Standard) 33215-2014 [33], GOST 33216-2014 [34], and Council Directive 2010/63 EU [35], «Guide for the Care and Use of Laboratory Animals». Eigth Edition (NRC, USA) [36].

### 5.6. Confocal Microscopy Method

To study the mechanism of action of the xi1E10 mAb, J774A.1 cells were used. One day prior to imaging, the cells were seeded at a density of 20,000 cells per well in an 8-well µ-slide (Ibidi, Gräfelfing, Germany). We used a lethal toxin consisting of recombinant subunits: PA (protective antigen) and fluorochrome-labeled FITC LF (lethal factor, LF-FITC), and a monoclonal antibody xi1E10 (Molecular Biology Laboratory, State Research Center of Applied Microbiology and Biotechnology).

To stain late endosomes and lysosomes, the following fluorochromes were used: Rab-7- (fluorescent dye, polyclonal antibodies to late endosome receptors Rab-7 with fluorochrome Alexa Fluor 647 (RRID: AB_198337, Gbiosciences, Maryland Heights, MO, USA, 2 µg/well)) and Lysotracker Red DND 99 (Invitrogen, Carlsbad, CA, USA, 75 μM/well), respectively. The nucleus of cells was stained with Hoechst 33258 (Sigma, USA, 1 µg/well) and the cytoplasm was stained with CellTracker (Invitrogen, USA, 5 μM/well).

To label LF with FITC fluorochrome, 600 μL of borate buffer (50 mM borate buffer with pH 8.5), 355 μL of LF (1.4 mg/mL) and 50 μL of FITC (1 mg/mL) were mixed and then left to incubate at room temperature (25 °C) for 2 h. After incubation, the reaction was stopped with Tris buffer solution (100 mM Tris buffer with pH 7.5). Dialysis against PBS was then performed to remove excess unbound FITC and replace the buffer.

For neutralization, lethal toxin was incubated with mAb xi1E10 for 1 h in a CO_2_ incubator at 37 °C. Then, either active or neutralized mAb xi1E10 lethal toxin was added to J774A.1 cell and incubated for 1.5 h in a CO_2_ incubator at 37 °C.

Microscopic observations and confocal imaging were performed using an OLYMPUS FV3000 scanning laser confocal microscope (OLYMPUS, Tokyo, Japan) with an oil immersion objective x60 (Objective Lens UPLXAPO60XO 60.0X/1.518 Oil, N/A 1.42, U-DIC60). The following lasers were used to visualize the samples: 405 nm (Hoechst 33258); 488 nm (FITC) and 640 nm (RAB-7, Lysotracker Red DND 99, CellTracker). The laser power was set to 10% of its maximum capacity. Colocalization of FITC (toxin) and Lysotracker Red DND 99 (lysosomes) fluorochrome signals in confocal images was estimated by the Pearson correlation coefficient using the Just Another Colocalisation Plugin (JACoP) for ImageJ 1.52v [37,38].

### 5.7. Statistical Analysis

Statistical analysis was carried out with GraphPad Prism 6.00 for Windows (GraphPad Prism Software Inc., San Diego, CA, USA) and Excel 2013 for Windows (Microsoft, Redmond, WA, USA).

Statistically significant differences in mouse survival between experimental groups were assessed using the Mantel-Cox log-rank test.

Dose–response curves were fitted using a four-parameter logistic nonlinear regression model in GraphPad Prism. The mAb concentrations were plotted on a logarithmic scale. The response at zero antibody concentration (Bottom) was constrained to the value corresponding to cell viability in the presence of LT only. IC50 values were obtained from the fitted curves as the antilogarithm of the LogIC50 parameter.

## Figures and Tables

**Table 1:** Comparison of therapeutic efficacy of monoclonal antibodies that recognize the same receptor-binding domain IV of PA.

mAb	Antibody Dose for 100% Protection	Reference
Raxibacumab (Abthrax^®^)	1.5 mg/kg in rat ^1^, 40 mg/kg in rabbit ^2^, 40 mg/kg in monkey ^3^	[13]
Obiltoxaximab ETI-204 (Anthim^®^)	4 mg/kg in rabbit ^2^	[14]
IQNPA	7.2 mg/kg in mouse ^4^	[10]
W1	0.021 mg/kg in rat ^1^, 1.6 mg/kg in mouse ^5^	[15]
Xi1E10	4.8 mg/kg in mouse	Our data

^1^ Fischer 344 rats were challenged with LT; ^2^ New Zealand white rabbit inhalational anthrax model with *B. anthracis* Ames spores; ^3^ Cynomolgus macaque inhalational anthrax model challenged with *B. anthracis* Ames spores. 90% protection at the dose indicated; ^4^ A/J mice were challenged with 24 LD_50_ of *B. anthracis* Sterne spores; ^5^ Unpublished data. A/J mice were challenged with 2 × 10^7^ Sterne spores (~1000 LD_50_). All PBS-treated mice died 48 h after challenge [21].

## Data Availability

The original contributions presented in this study are included in the article. Further inquiries can be directed to the corresponding author.
